# Clinical Heterogeneity in Inguinal Hernia Repair and the Need for Tailored Management: A Retrospective Observational Study of Postoperative Complications and Hospitalization Duration

**DOI:** 10.3390/jcm15031258

**Published:** 2026-02-05

**Authors:** Jeong Hee Han, Jung Bum Choi, Min Ju Kim, Jun Hyung Bang, Hong Jae Jo, Eun Ji Park, Byoung Chul Lee

**Affiliations:** 1 Department of Surgery, Biomedical Research Institute, Pusan National University Hospital, Busan 49241, Republic of Korea; ilovehers@naver.com (J.H.H.); kamata82@hanmail.net (J.B.C.); minju.kim712@gmail.com (M.J.K.); jhbang@pnuh.co.kr (J.H.B.); j1000h@nate.com (H.J.J.); 2 Department of Surgery, School of Medicine, Pusan National University, Busan 50612, Republic of Korea; 3 Department of Anesthesia and Pain Medicine, School of Medicine, Pusan National University, Busan 50612, Republic of Korea; eangelj1@naver.com; 4 Department of Anesthesia and Pain Medicine, Biomedical Research Institute, Pusan National University Hospital, Busan 49241, Republic of Korea

**Keywords:** inguinal hernia, clinical judgment, individualized medicine

## Abstract

**Background/Objectives:** The study aims to provide a comprehensive understanding of personalized treatment for patients with inguinal hernias at our hospital, focusing on complications, recurrence rates, and hospitalization duration to optimize treatment outcomes. **Methods:** Our center performs inguinal hernia surgery using an algorithm tailored to individual clinical conditions, developed in collaboration with the anesthesiology department. We retrospectively reviewed outcomes of open, totally extraperitoneal (TEP), and transabdominal preperitoneal (TAPP) approaches, with all procedures performed by a single surgeon. **Results:** A total of 229 patients (213 males; age range, 24–92 years; median age, 69 years) underwent inguinal hernia repair at Busan National University Hospital between January 2018 and April 2024. Patients in the open group had higher age and comorbidity burden (age/ASA American Society of Anesthesiologists physical status classification: open 74/3.5 vs. TAPP 70/2.0 vs. TEP 68/2.0; *p* = 0.036/< 0.001). There were no statistically significant differences in intraoperative complications (*p* = 1.000); however, the conversion rate was slightly higher in the TEP group (TEP 2 vs. TAPP 1). Length of hospital stay was longest in the TAPP group (open 3.77 days vs. TAPP 3.98 days vs. TEP 3.27 days; *p* = 0.817), while postoperative complication rates did not differ significantly among groups (overall complications: open 15.4% vs. TAPP 6.2% vs. TEP 4.3%; *p* = 0.100). **Conclusions:** Laparoscopic surgery is recommended when general anesthesia is feasible, with TEP preferred for patients without previous surgeries and TAPP for those with preperitoneal space (PPS) access challenges due to previous surgeries or radiation therapy. Open surgery is suitable for patients unable to undergo general anesthesia. Anesthesia and surgical approaches should be based on patient preferences and individual clinical conditions.

## 1. Introduction

Inguinal hernia repair is one of the most commonly performed surgeries in general surgery and is an essential procedure, included as a required skill for general surgery residents in Korea [1]. Globally, groin hernia repair is performed on a very large scale, and international guidelines highlight its substantial population-level burden and impact on healthcare systems [2,3]. As medical advancements extend life expectancy and the elderly population grows, the number of older patients with hernias is expected to increase. These patients often have multiple underlying health conditions and slower recovery rates, which heighten the risks associated with surgery. Beyond perioperative risk, inguinal hernias can cause pain, functional limitation, and reduced quality of life, and may progress or become complicated over time; importantly, surgery is the only definitive treatment that corrects the abdominal wall defect, whereas non-operative strategies do not provide a cure [2,3]. However, many still choose surgery due to their active lifestyles and the importance they place on quality of life [2,4]. Given these trends, it is critical to establish safer surgical techniques and anesthesia methods that lower complication and recurrence rates.

Although guidelines for hernia repair exist, no single standard recommendation applies to all inguinal hernia cases [2,3]. In open surgery, the Lichtenstein method is widely used, while laparoscopic techniques, such as TEP and TAPP, are generally preferred. Studies have evaluated both approaches, showing no significant differences in complication rates. However, laparoscopic surgery is generally recommended over open surgery due to quicker recovery times and a lower risk of chronic pain [2,5]. Despite this, existing research and guidelines lack clarity on how to select the most appropriate repair method for patients with specific clinical factors, such as prior abdominal surgeries, prostate surgery, or anesthesia type. This gap in the literature leaves surgeons without clear guidance for tailoring surgical strategies to individual patients, particularly in a rapidly aging society. This study aims to evaluate the safety and effectiveness of inguinal hernia repair using a personalized treatment algorithm that integrates current hernia guidelines with the patient’s clinical context. Moreover, it emphasizes the importance of patient-centered clinical decision-making, offering evidence-based solutions to meet the increasing demands of an aging society with complex medical needs.

## 2. Materials and Methods

### 2.1. Operation Method

**Figure 1:**
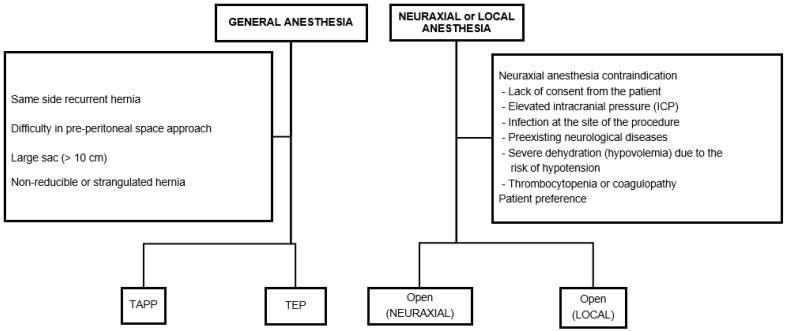
Algorithm for selecting the surgical approach for inguinal hernia repair according to anesthesia type and clinical conditions. TAPP transabdominal preperitoneal repair, TEP totally extraperitoneal repair.

Three surgical approaches were utilized: open, TEP, and TAPP (Figure 1). Open surgery involved the Lichtenstein operation and was primarily performed for patients for whom general anesthesia posed difficulties. The decision regarding spinal anesthesia or local anesthesia was primarily determined based on patient preference and consultation with anesthesiologists, particularly for patients with challenges undergoing general anesthesia. The preferred laparoscopic surgical approach was the TEP technique. However, TAPP surgery was performed in the following situations:

1. Recurrent hernia where the previous surgical approach was anterior
2. PPS for TEP surgery was challenging due to previous lower abdominal surgery, robotic-assisted radical prostatectomy, cystectomy, and radiation therapy
3. Large sac or mass exceeding 10 cm
4. Manual reduction of the hernia was not possible
5. Strangulated hernia

### 2.2. Anesthesia Method

If possible, surgery was performed under general anesthesia using laparoscopy. For patients with severe pulmonary disease or significantly reduced lung function as determined by preoperative pulmonary function tests, neuraxial anesthesia should be considered. Additionally, for patients with cardiac conditions such as heart failure, severe valvular heart disease, left ventricular outflow obstruction, etc., there are concerns about sympathetic nerve blockade and hemodynamic instability. Therefore, the method of anesthesia and surgery was determined after consulting with the anesthesiologist to evaluate the risks associated with each type of anesthesia [6,7]. Neuraxial anesthesia can be a safe alternative for elderly patients who are at high risk for general anesthesia. However, if there are contraindications to neuraxial anesthesia, the surgery was performed under local anesthesia.

### 2.3. Patient Population

This retrospective observational study included 229 patients who underwent surgery after being diagnosed with inguinal hernia at Busan National University Hospital from October 2018 to January 2023. A single surgeon performed all surgeries. All patients underwent two follow-up observations within 1–2 weeks and one month after surgery. Subsequent follow-up was conducted based on the occurrence of complications.

### 2.4. Main Outcome

Data were collected through electronic medical records, and the postoperative outcomes were analyzed based on surgical techniques. The length of stay was calculated starting from the first preoperative day of surgery (postoperative day 1; POD1). Intraoperative bleeding was defined as excessive if the estimated blood loss (EBL) exceeded 100 cc. Chronic pain was defined as pain that persisted for at least 6 months after surgery.

### 2.5. Statistical Analysis

Patient’s characteristics and group comparisons are presented in Table 1, Table 2 and Table 3. Continuous variables are presented as means and standard deviations or medians and interquartile ranges, based on satisfaction of the normality assumption, which was tested using the Shapiro–Wilk test. Categorical variables are presented as frequencies and percentages. The chi-squared or Fisher’s exact test was used to analyze categorical variables, and the independent t-test or Wilcoxon rank-sum test was used for continuous variables. No imputation methods were applied for missing data. Statistical analysis was performed using the language R version 4.3.3 (R Core Team, 2024, http://cran.r-project.org). A *p* value of <0.05 was considered statistically significant.

## 3. Results

### 3.1. Preoperative Data of Patients

Preoperative patient variables are presented in Table 1. Among all groups, the average age (74 years) and average ASA score (3.50) were the highest in the open surgery group. The average age was higher (70 years) in the TAPP group than in the TEP group (68 years). The ASA score was the same (2.0) for both the TAPP and TEP groups. The gender distribution showed a higher proportion of male patients (93% vs. 7%).

### 3.2. Intraoperative Data of Patients

**Figure 2:**
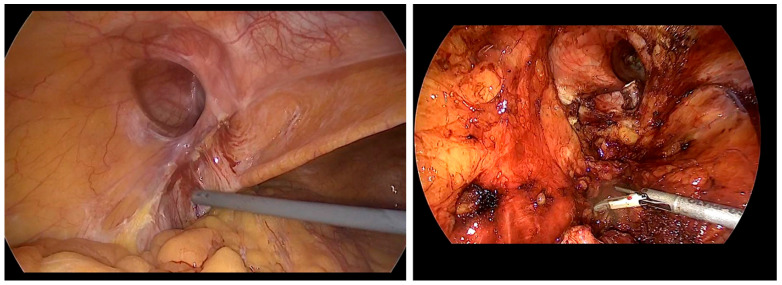
Small bowel adhesions caused by previous abdominal surgery are observed on the (**left**) image. After the adhesions were removed, a left inguinal hernia was identified on the (**right**) image.

**Figure 3:**
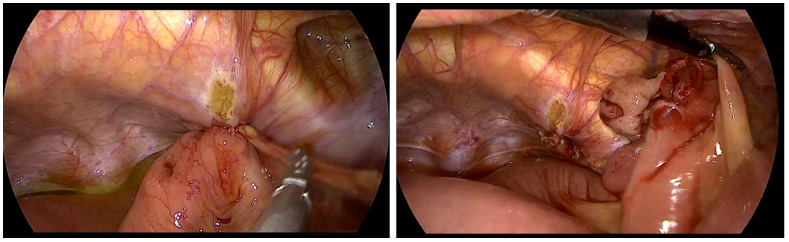
The small bowel herniated through the obturator canal, resulting in necrosis (**left**). The herniated bowel was successfully reduced (**right**).

Intraoperative data of patients are presented in Table 2. Conversion of surgery methods was needed in three cases: one case involved injury to the sigmoid colon (TEP → low midline incision), another was due to severe adhesions from previous surgeries (Figure 2; TEP → TAPP), and the third case required small bowel resection due to necrosis of the herniated bowel (Figure 3; TAPP → low midline incision).

### 3.3. Postoperative Data of Patients

Postoperative data of patients are presented in Table 3. The overall complication rate was 6.1%. The most common complications were seroma and urinary retention, each occurring at a rate of 1.7%. There was no significant difference in complication rates between the surgical techniques.

## 4. Discussion

Herniorrhaphy is one of the most commonly performed surgeries worldwide [4]. Age is a significant risk factor, with the highest incidence observed in individuals aged 70–80 years. Given the global trend of an aging population, the number of hernia patients is expected to increase [2]. In light of this rising incidence, it is crucial to ensure the safety of patients and minimize both complications and recurrence rates by providing individualized, patient-tailored surgical approaches, particularly for elderly patients and those with comorbidities [5]. This study represents the first reported case of customized surgery based on the guidelines established by the HerniaSurge group. The overall complication rate following inguinal hernia repair surgery is generally reported to be approximately 8–10%, with the most common complications being hemorrhage (0.86%) and wound infection (0.48%) [4]. Although surgical site infection (SSI) after elective inguinal hernia repair is generally uncommon, SSI remains clinically important in elderly patients and those with comorbidities, particularly in the setting of mesh implantation, because even minor infection can delay recovery and increase healthcare utilization. Recently, inflammation-related biomarkers have been explored to support early risk stratification of postoperative infection. In colorectal surgery cohorts, early postoperative butyrylcholinesterase (BChE) has been reported to correlate with SSI risk and severity, suggesting potential value as a readily obtainable marker of postoperative inflammatory burden. Although these data cannot be directly extrapolated to groin hernia repair, future studies may evaluate whether incorporating biomarkers such as BChE could improve SSI surveillance in high-risk hernia patients [8]. In our institution, by adhering to the proposed algorithm (Online Resource 1), the overall complication rate observed among surgical patients was 6.1%, with no complications classified above Clavien-Dindo grade II, and, to date, no cases of hernia recurrence have been observed. This tailored surgical approach is expected to enhance patient safety and result in a lower incidence of complications.

**Figure 4:**
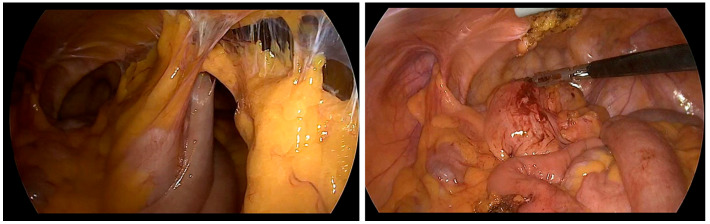
Small bowel adhesions caused by previous abdominal surgery are shown in the (**left**) image. After removal of the adhesions, a left inguinal hernia is visible in the (**right**) image.

For patients who could undergo general anesthesia, laparoscopic surgery was prioritized, with TEP repair being preferred as the initial approach. Although no significant differences in complication rates were observed between open and laparoscopic surgeries, the laparoscopic approach demonstrated lower rates of chronic pain and faster recovery to normal daily activities [2,3]. In elderly or comorbid patients, postoperative pain control is crucial for early ambulation and recovery while minimizing opioid-related adverse effects. A study comparing three postoperative analgesic regimens after open inguinal hernia repair reported lower postoperative pain scores with multimodal strategies than with acetaminophen monotherapy, supporting an opioid-sparing approach when feasible [9]. There is no definitive evidence to suggest one laparoscopic method is superior to the other, and no differences were found in complication rates between the two techniques. Generally, TEP has a relatively higher risk of bleeding and conversion rate, whereas TAPP repair carries a greater risk of intra-abdominal organ injury [2,6,10]. In our center, no significant difference in complication rates was observed between TEP and TAPP. However, TAPP had a relatively higher incidence of urinary retention and longer hospital stays. Both TEP and TAPP are considered safe due to their low complication rates. However, TEP is often favored as the first-line treatment because it involves a shorter hospital stay and avoids peritoneal damage, thus reducing the risk of bowel injury and intra-abdominal adhesions [7,11,12,13]. From a technical standpoint, TEP may be challenging when the preperitoneal working space cannot be safely created or maintained, such as in patients with dense scarring from prior lower abdominal surgery; peritoneal tears can also compromise the workspace and increase the likelihood of conversion [2,3,7,11,12,13]. In contrast, TAPP provides a wider operative field and direct intraperitoneal visualization, which can facilitate dissection in complex or incarcerated cases, but it requires peritoneal entry and adhesiolysis and therefore may increase the risk of visceral injury, demanding advanced laparoscopic expertise and careful patient selection [2,3,6,7,10,11,12,13]. In cases where access to the PPS was difficult during TEP, TAPP was selected as the alternative approach (Figure 2, severe adhesions due to previous operation) [14,15]. The guidelines from the HerniaSurge group were followed for recurrent hernias. If the initial surgery used an anterior approach, a posterior approach using TAPP was performed. However, if the previous surgery involved a posterior approach, TEP was selected. The superiority of either approach has not been conclusively established, and the choice of technique should be tailored to the surgeon’s expertise [16]. In cases of incarcerated hernias or patients who have undergone multiple surgeries due to recurrence, where extensive adhesions between the hernia sac and bowel may be present, TAPP allows for direct visualization of the bowel, making it a potentially safer technique for surgeons skilled in advanced laparoscopic procedures (Figure 3, incarcerated hernia, pre- and post-reduction; Figure 4, bowel adhesion from a previous low midline incision). Accordingly, Figure 4 is intended to illustrate a technical scenario in which bowel assessment and adhesiolysis under direct visualization may be required, rather than to imply an absolute algorithmic preference for one approach.

For patients who could not undergo general anesthesia, the open method was used with either local or spinal anesthesia. In open surgery, the Prolene Hernia System repair was initially attempted. If access to the PPS was difficult due to previous surgeries or radiation therapy, the Lichtenstein technique was used instead. Both techniques were chosen because they have been reported to result in fewer postoperative complications and lower recurrence rates compared to other open methods [17,18]. While laparoscopic surgery under general anesthesia is generally preferred for patients, the risks associated with it often outweigh the benefits in high-risk cases [16,19,20,21]. There is controversy about using spinal versus local anesthesia when utilizing the open surgical method. Many studies suggest that local anesthesia is preferred because it has fewer side effects and is less costly compared to spinal anesthesia [22,23]. However, at our center, spinal anesthesia was prioritized to ensure a stable surgical environment and to address patient concerns about pain and anxiety. For patients who could not receive spinal anesthesia, the open method was performed under local anesthesia [24,25].

This study demonstrated that applying a tailored surgical approach for elderly patients and those with comorbidities significantly improved safety and reduced complication rates. However, as this study was conducted at a single institution with a relatively small patient cohort, larger multicenter studies are required to validate these findings. The inherent risk of bias due to the study design, such as recall bias and the lack of randomization, should also be considered. Second, the small sample size may limit the study’s statistical power and potentially underestimate recurrence and complication rates. Additionally, since the study was performed on a specific patient group at a single institution, the findings may not be generalizable to other populations or institutions. It will be essential to assess the reproducibility of the results when other surgeons follow the same protocol. Such research will help clarify the effectiveness of a tailored approach and establish the optimal treatment methods for diverse patient populations. Despite these limitations, this study is significant as it represents the first attempt to apply a personalized hernia treatment approach based on individual clinical circumstances. Future studies should also evaluate long-term outcomes, including recurrence rates and chronic pain, and provide a more detailed analysis of the differences in recovery and complications between local and spinal anesthesia. Finally, studies comparing the benefits of laparoscopic versus open surgical methods in high-risk patients are needed.

## 5. Conclusions

In this single-center retrospective cohort of 229 patients undergoing inguinal hernia repair using a patient-tailored algorithm that integrates anesthetic feasibility and key clinical factors, short-term outcomes were acceptable: the overall complication rate was 6.1%, with no events exceeding Clavien–Dindo grade II; SSI occurred in 1.3%, and conversion was required in 1.3%. No recurrences or chronic pain were documented during the available follow-up. Overall, these findings suggest that individualized approach selection (open vs. TEP vs. TAPP), consistent with contemporary guideline principles, can be implemented with a low short-term complication burden in elderly and comorbid patients. Larger multicenter studies with standardized follow-up are warranted to confirm long-term durability (recurrence and chronic pain) and to better delineate which patient subgroups benefit most from specific operative and anesthetic strategies [2,3].

## Figures and Tables

**Table 1:** Pre-operative data of patients.

	Overall	OPEN	TAPP	TEP	*p*	Test
*n*	229	26	65	138		
AGE (median [IQR])	69.00 [62.00, 76.00]	74.00 [69.00, 78.75]	70.00 [63.00, 75.00]	68.00 [59.25, 76.00]	0.036	†
SEX (%)					0.866	‡
Female (%)	16 (7.0)	2 (7.7)	5 (7.7)	9 (6.5)		
Male (%)	213 (93.0)	24 (92.3)	60 (92.3)	129 (93.5)		
ASA (median [IQR])	2.00 [2.00, 3.00]	3.50 [3.00, 4.00]	2.00 [2.00, 3.00]	2.00 [2.00, 3.00]	<0.001	†
ANESTHESIA (%)					<0.001	
LOCAL	10 (4.4)	10 (38.5)	0 (0.0)	0 (0.0)		
NEURAXIAL	16 (7.0)	16 (53.8)	0 (0.0)	0 (0.0)		
GENERAL	203 (88.6)	0 (0.0)	65 (100.0)	138 (100.0)		
DM (%)	51 (22.4)	7 (28.0)	13 (20.0)	31 (22.5)	0.716	‡
HTN (%)	95 (41.7)	15 (57.7)	31 (47.7)	49 (35.8)	0.058	‡
LC (%)	10 (4.4)	4 (15.4)	0 (0.0)	6 (4.3)	0.006	‡
COPD (%)	20 (8.7)	13 (50.0)	1 (1.5)	6 (4.3)	<0.001	‡
ASTHMA (%)	15 (6.6)	6 (23.1)	2 (3.1)	7 (5.1)	0.005	‡
CVA (%)	22 (9.6)	6 (23.1)	3 (4.6)	13 (9.4)	0.032	‡
ANGINA, MI (%)	29 (12.7)	5 (19.2)	8 (12.3)	16 (11.6)	0.536	‡
HEART FAILURE (%)	7 (3.1)	6 (23.1)	0 (0.0)	1 (0.7)	<0.001	‡
CRF (%)	19 (8.3)	5 (19.2)	3 (4.6)	11 (8.0)	0.085	‡
RECUR (%)	15 (6.6)	1 (3.8)	9 (13.8)	5 (3.6)	0.024	‡

ASA American Society of Anesthesiologists physical status classification, DM diabetes mellitus, HTN hypertension, LC liver cirrhosis, COPD chronic obstructive pulmonary disease, CVA cerebrovascular accident, MI myocardial infarction, CRF chronic renal failure. †: Wilcoxon rank sum test. ‡: Fisher’s exact test.

**Table 2:** Intra-operative data of patients.

	Overall	OPEN	TAPP	TEP	*p*	Test
*n*	229	26	65	138		
Bleeding (%)	0 (0.0)	0 (0.0)	0 (0.0)	0 (0.0)		‡
Bowel injury (%)	0 (0.0)	0 (0.0)	0 (0.0)	1 (0.7)	1.000	‡
conversion (%)	3 (1.3)	0 (0.0)	1 (1.5)	2 (1.4)	1.000	‡

‡: Fisher’s exact test.

**Table 3:** Post-operative data of patients.

	Overall	OPEN	TAPP	TEP	*p*	Test
*n*	229	26	65	138		
HD	3.53	3.77	3.98	3.27		
Total (%)	14(6.1)	4(15.4)	4(6.2)	6(4.3)	0.100	‡
Hematoma (%)	2 (0.9)	1 (3.8)	1 (1.5)	0 (0.0)	0.077	‡
Seroma (%)	4 (1.7)	1 (3.8)	2 (3.1)	1 (0.7)	0.202	
Surgical site infection (%)	3 (1.3)	1 (3.8)	1 (1.5)	1 (0.7)	0.202	‡
Urinary retention (%)	4 (1.7)	1 (3.8)	3 (2.2)	0 (0.0)	0.302	‡
Ileus (%)	1 (0.4)	0 (0.0)	0 (0.0)	1 (0.7)	1.000	‡
Chronic pain (%)	0 (0.0)	0 (0.0)	0 (0.0)	0 (0.0)		‡
RECUR (%)	0 (0.0)	0 (0.0)	0 (0.0)	0 (0.0)		‡

HD hospital day. ‡: Fisher’s exact test.

## Data Availability

The data presented in this study are not publicly available due to privacy and ethical restrictions but are available from the corresponding author upon reasonable request.

## Abbreviations

The following abbreviations are used in this manuscript:

TEP	Totally Extra Peritonea
TAPP	Trans Abdominal Pre Peritoneal
PPS	Pre Peritoneal Space
ASA	American Society of Anesthesiologists’ physical status classification
DM	Diabetes Mellitus
HTN	Hypertension
LC	Liver Cirrhosis
COPD	Chronic Obstructive Pulmonary Disease
CVA	Cerebro Vascular Accident
MI	Myocardial Infarction
CRF	Chronic Renal Failure
